# Broad-Spectrum Antibacterial Activity of Antioxidant Octyl Gallate and Its Impact on Gut Microbiome

**DOI:** 10.3390/antibiotics13080731

**Published:** 2024-08-04

**Authors:** Junshu Yang, Trevor J. Gould, Byeonghwa Jeon, Yinduo Ji

**Affiliations:** 1 Department of Veterinary and Biomedical Sciences, College of Veterinary Medicine, University of Minnesota, Saint Paul, MN 55108, USA; 2 Minnesota Supercomputing Institute, University of Minnesota, Minneapolis, MN 55455, USA; 3 Division of Environmental Health Sciences, School of Public Health, University of Minnesota, Saint Paul, MN 55108, USA

**Keywords:** antioxidant octyl gallate, antibacterial activity, *Staphylococcus aureus*, gut microbiome

## Abstract

In this study, we investigated the antibacterial activity of octyl gallate (OG), an antioxidant food additive, against both Gram-positive and Gram-negative bacterial pathogens. OG demonstrated robust bactericidal activity against Gram-positive bacterial pathogens with minimum inhibitory concentrations (MIC) of 4 to 8 µg/mL and minimum bactericidal concentrations (MBC) of 8 to 16 µg/mL in vitro. However, OG exhibited limited antibacterial activity against Gram-negative bacteria, including *E. coli*, although it could inhibit bacterial growth in vitro. Importantly, OG administration in mice altered the fecal microbiome, significantly reducing microbial diversity, modifying community structure, and increasing the abundance of beneficial bacteria. Additionally, OG displayed low cytotoxicity and hemolytic activity. These findings suggest that OG could be developed as a novel antibacterial agent, particularly against multi-drug-resistant MRSA. Our results provide new insights into the therapeutic potential of OG in modulating the gut microbiome, especially in conditions associated with microbial imbalance, while ensuring food safety.

## 1. Introduction

*Staphylococcus aureus* poses significant health threats to both humans and animals, also serving as a major foodborne pathogen [1]. Particularly, the rise of multi-drug-resistant *S. aureus* strains, particularly methicillin-resistant *S. aureus* (MRSA) variants like hospital-acquired MRSA (HA-MRSA), community-acquired MRSA (CA-MRSA), and livestock-associated MRSA, has become a pressing concern in public health [2,3]. Therefore, the development of new classes of antibacterials is crucial to confront the escalating crisis of antibiotic resistance. As bacteria evolve and acquire resistance to current antibiotics, the efficacy of existing treatments diminishes, resulting in higher morbidity and mortality rates and increased healthcare costs globally [4] (Centers for Disease Control and Prevention (CDC), 2019). Recognizing antibiotic resistance as a top ten global public health threat, the World Health Organization (WHO) underscores the urgent need for innovative antibacterial agents [5] (WHO, 2020). Novel antibacterials with unique mechanisms of action are essential to stay ahead of bacterial adaptation and ensure effective treatment options for future generations. Developing these new classes of antibacterials can play a critical role in combating multi-drug-resistant pathogens and protecting public health. 

Octyl gallate (OG), a derivative of gallic acid, exhibits promising antibacterial activity against both Gram-positive and Gram-negative bacteria, including *Salmonella choleraesius, Bacillus subtilis*, and *S. aureus* [6,7,8,9]. Additionally, OG is an antioxidant food additive approved by the US Food and Drug Administration, making it a compound of interest for the development of new antimicrobial agents. Recent studies have shown that OG can synergize with conventional antibiotics, enhancing their efficacy against various resistant bacterial strains [10,11,12,13]. This synergistic effect not only broadens the antibacterial spectrum of existing drugs but also potentially reduces required doses, thereby minimizing side effects. Ongoing research underscores OG’s potential as a potent antibacterial agent, particularly significant in the face of escalating antibiotic resistance.

Moreover, OG has emerged as a significant compound in combating bacterial biofilms, which are resilient communities of bacteria adhering to surfaces and notorious for their resistance to conventional antibiotics. Recent research highlights OG’s substantial anti-biofilm activities, disrupting biofilm formation and maintenance across various bacterial species [14,15,16,17]. OG enhances the susceptibility of MRSA biofilms to bacitracin, effectively reducing biofilm biomass and bacterial viability [18]. Moreover, OG inhibits biofilm formation in *Pseudomonas aeruginosa* by interfering with quorum sensing, a crucial regulatory system in biofilm development [14]. In addition, OG can potentiate photodynamic inactivation systems to eradicate established biofilm of *P. aeruginosa* [16,17]. These findings underscore OG’s potential in preventing biofilm-related infections and augmenting the effectiveness of existing antibiotic therapies. OG not only prevents biofilm establishment but also increases the vulnerability of existing biofilms to antibiotics, thereby significantly mitigating biofilm-related antibiotic resistance. These dual actions of OG, as both an antibacterial and an anti-biofilm agent, position it as a promising candidate for combatting MRSA infections, especially those complicated by biofilm-related antibiotic resistance.

Our previous studies have demonstrated that OG can significantly enhance the antimicrobial and anti-biofilm activities of β-lactams and bacitracin against multi-drug-resistant MRSA and *S. epidermidis*. In this study, we examined the antibacterial activity of OG against various *S. aureus* isolates, including HA-MRSA, CA-MRSA, LS-MRSA, and MSSA, as well as *S. epidermidis*, *Streptococcus pyogenes*, *B. subtilis*, *E. coli*, *Acinetobacter baumannii*, *Klebsiella pneumoniae*, and *P. aeruginosa*. Additionally, we investigated the impact of OG on the gut microbiome upon consumption. Moreover, we assessed the cytotoxicity of OG using human normal foreskin fibroblast HFF, human lung cancer epithelial A549 cells, and sheep red blood cells. Our results provide new insights into the antibacterial properties of OG. 

## 2. Results

### 2.1. OG Possesses Potent Antibacterial Activity against Gram-Positive Bacteria

**Figure 1:**
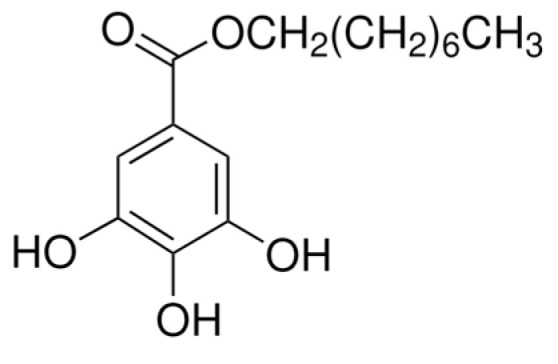
Structure of octyl gallate (OG) with ≥99.0% (HPLC).

It has been reported that OG exhibits antibacterial and antibiofilm activity against different bacteria, including *S. aureus* [8,9,10,11,12]. To further validate the antibacterial activity of OG, we examined the antibacterial profiles of OG (Figure 1) and found that OG possesses potent bactericidal activity against various Gram-positive bacteria, including multi-drug-resistant HA-MRSA, CA-MRSA, LA-MRSA, *S. epidermidis*, *S. pyogenes*, and *B. subtilis* with a minimum inhibitory concentration (MIC) of 4 to 8 µg/mL and a minimum bactericidal concentration (MBC) ranging from 8 to 16 µg/mL (Table 1). However, OG exhibited poor antibacterial activity against *E. coli*, *A. baumannii*, *K. pneumoniae*, and *P. aeruginosa* with an MIC and an MBC of more than 64 or 128 µg/mL (Table 1 and Table S1).

**Figure 2:**
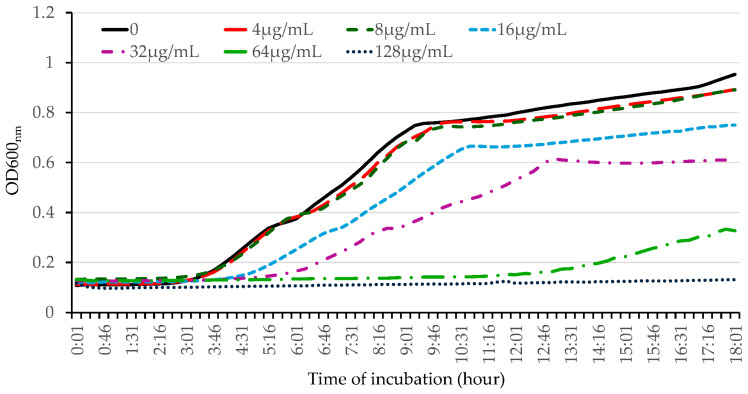
Kinetic determination of the effect of OG on the growth of *E. coli*. The overnight bacterial culture was inoculated at 1% into fresh LB medium, and different concentrations of OG were added. Bacterial growth was determined kinetically by measuring the optical density every 15 min for 18 h at 37 °C using a 96-well plate reader. The experiment was repeated three times, producing similar results.

To further evaluate the antibacterial activity of OG against Gram-negative bacteria, we used *E. coli* as a model bacterium. We performed kinetic studies and examined the impact of OG on bacterial growth by measuring the optical density of the bacterial culture. OG inhibited bacterial growth in a dose-dependent manner at concentrations less than 128 µg/mL and abolished bacterial growth at concentrations higher than 128 µg/mL (Figure 2 and Table S1).

### 2.2. OG Possesses Robust Bactericidal Activity against HA-MRSA

**Figure 3:**
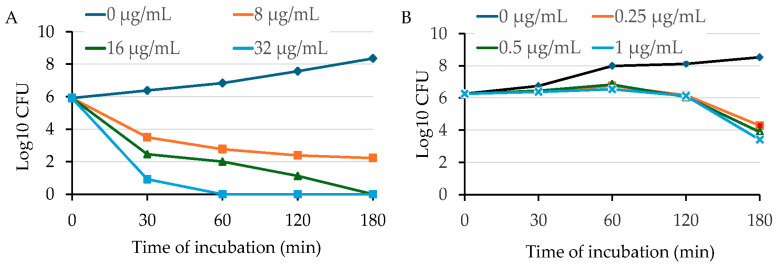
Time-dependent killing of HA-MRSA by OG (**A**) or triclosan (**B**). HA-MRSA WCUH29 was grown in MHB2 to early log phase, and the bacterial cells were exposed to different concentrations of OG. An aliquot of culture was taken from each treatment group at different time points after exposure to OG or triclosan, followed by a serial dilution. The diluted culture was plated onto TSA and incubated at 37 °C. The values of log_10_ CFU/mL were calculated, and the results are representative of three independent experiments.

To further validate the bactericidal activity of OG against MRSA, we conducted time-killing assays by adding different concentrations (1 × MIC, 2 × MIC, and 4 × MIC) of OG into the early exponential phase of *S. aureus* cultures. OG showed potent bactericidal activity against HA-MRSA WCUH29 in a dose-dependent manner (Figure 3A). The number of surviving bacterial cells dramatically decreased by more than 3 log_10_ CFUs 30 min after adding OG at the MBC level of 16 µg/mL (2 × MIC) compared with the negative ethanol control (Figure 3A). The positive control, triclosan, remarkably reduced the count by 3 log_10_ CFUs after 3 h of exposure to more than 2 × MIC of Triclosan compared to the negative control (Figure 3B). Importantly, adding 2 to 4 × MIC of OG eliminated bacterial survival after 3 h of exposure, reducing the count by approximately 6 log_10_ CFUs (Figure 3A). In contrast, triclosan killed the staphylococci at 4 to 8 × MIC after 18 h of exposure. 

### 2.3. OG Enhances the Cell Permeability of HA-MRSA and E. coli

**Figure 4:**
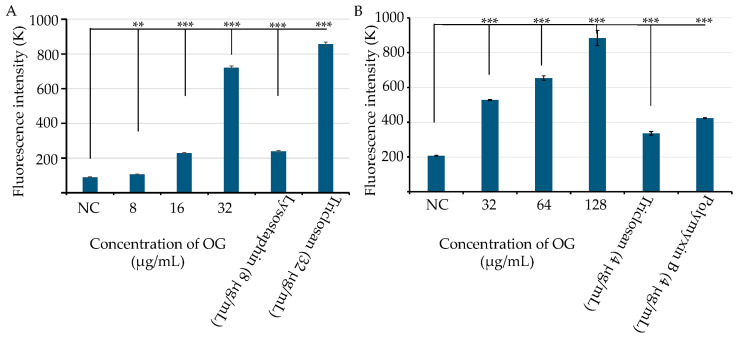
Effects of octyl gallate (OG) on the cell permeability in *S. aureus* WCUH29 (HA-MRSA) and *E. coli*. (**A**) The cell permeability of *S. aureus* WCUH29 (HA-MRSA) was assessed after exposure to various concentrations of OG using the fluorescent probe propidium iodide (PI; 10 µg/mL). Lysostaphin and triclosan served as positive controls. (**B**) The permeability of *E. coli* was evaluated under similar conditions, with triclosan and polymyxin B as positive controls. Fluorescence intensity was measured using a BioTek Synergy II spectrophotometer (Agilent, Santa Clara, CA, USA) with 535 nm excitation and 590 nm emission, in a black 96-well plate. Data represent the averages and standard deviations of fluorescence values from three samples per condition in a single experiment. The experiment was repeated at least three times. NC: bacterial cells plus PI as a negative control. Differences in fluorescence intensity between the negative control and treatment groups were statistically analyzed using Student’s t-test. The symbols ** and *** indicate *p* values < 0.01 and < 0.001, respectively.

Previous studies have demonstrated that OG can selectively enhance the antibacterial activity of penicillin and bacitracin by increasing the access of these antimicrobials to their cellular targets, likely through altering the cell wall permeability of *S. aureus* and *S. epidermidis* [11,12,18]. Based on these findings, we hypothesized that OG plays a role in enhancing the bacterial cell permeability. To test this, we exposed MRSA and *E. coli* to different concentrations of OG along with a fluorescent probe, propidium iodide (PI). OG significantly increased the fluorescence intensity in a dose-dependent manner, indicating OG increased permeability in both *S. aureus* (Figure 4A) and *E. coli* (Figure 4B). As expected, the positive controls, lysostaphin (8 µg/mL) or Triclosan (32 µg/mL), and Triclosan (4 µg/mL) or polymyxin B (4 µg/mL) significantly increased the permeability of *S. aureus* (Figure 4A) and *E. coli* (Figure 3B), respectively.

### 2.4. OG Has Low Cytotoxicity and Hemolytic Activity

**Figure 5:**
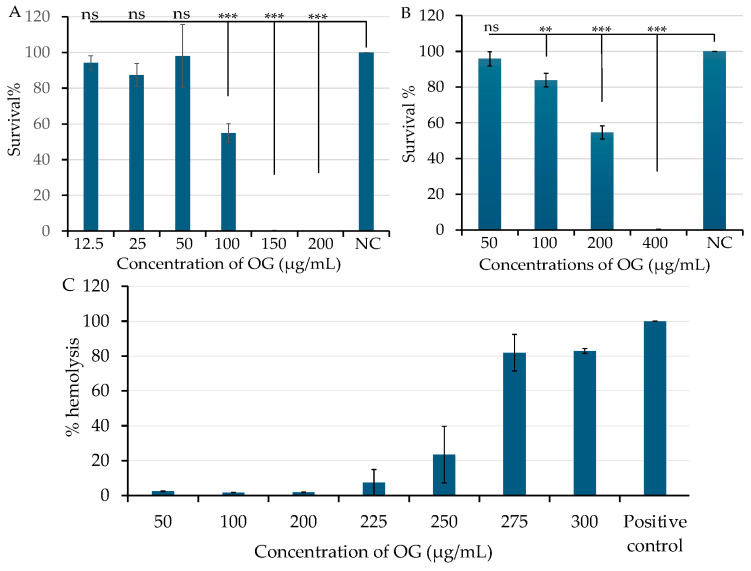
Effect of octyl gallate (OG) on human cell viability and hemolytic activity in sheep red blood cells. Human normal fibroblast HFF cells (**A**) and human lung cancer epithelial A549 cells (**B**) were exposed to various concentrations of OG. Negative control cells were treated with DMSO as a vehicle control. Cell viability was measured after 24 h of treatment using a BioTek Synergy II spectrophotometer, as described in Section 4.6. The symbols ** and *** indicates a significant difference between the control and treated cells at *p* < 0.01 and *p* < 0.001, respectively. No significant difference is indicated as “ns”. Hemolytic activity of OG was assessed in sheep red blood cells (**C**), with standard saponin serving as the positive control and PBS as the negative control. Hemolysis (%) was calculated using the following formular: [(A450 test sample − A450 negative control)/(A450 positive control − A450 negative control)] × 100. Data represent the average of three samples per condition in a single experiment. Each experiment was repeated at least three times.

Although OG is an FDA-approved antioxidant used as a food additive, we further evaluated its cytotoxicity. Cytotoxicity assays were conducted using normal human foreskin fibroblast HFF cells and human lung cancer epithelial A549 cells in triplicate experiments, as previous described [22]. The human cells were exposed to varying concentrations of OG for 24 h. Cell viability was measured as described in Section 4.6, with DMSO serving as a negative vehicle control. Over 95% of HFF (Figure 5A) and A549 cells (Figure 5B) remained viable after exposure to OG at 50 µg/mL (177.09 μM). However, exposure to 150 µg/mL and 400 µg/mL of OG resulted in more than 99% cell death in HFF and A549 cells, respectively (Figure 5A,B). Additionally, less than 2% sheep red blood cells were lysed following exposure to OG concentrations ranging from 50 to 200 µg/mL (Figure 5C). These findings collectively indicate the low cytotoxicity of OG. 

### 2.5. Feeding OG Affects the Diversity of Gut Microbiome of C57BL/6 Mice

**Figure 6:**
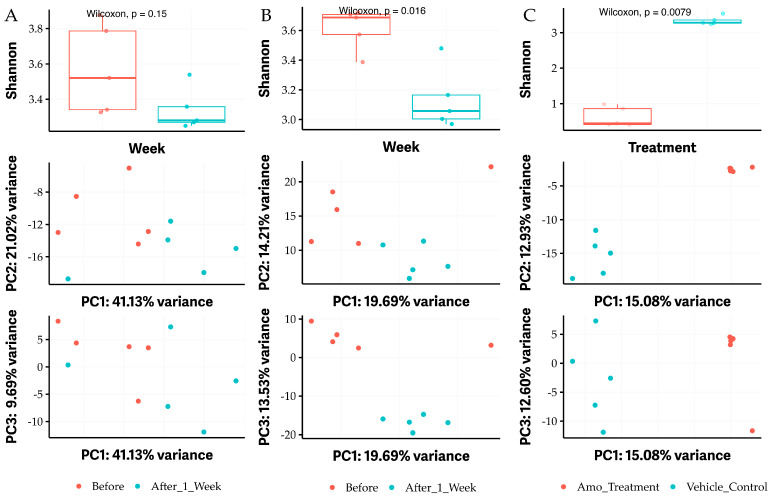
The alpha diversity of fecal microbiome in mice affected by administrating OG. (**A**) The negative vehicle control (VC) group between before treatment (blue color) and after 1 week treatment with VC (red color) with Shannon statistical analysis of alpha-diversity (top) and PC variance (middle and bottom). (**B**) Shannon alpha-diversity significantly affected between before treatment (blue color) and after 1 week treatment with 50 mg/kg body weight twice a day OG (red color) (top) and PC variance (middle and bottom). (**C**) Shannon alpha-diversity significantly affected between vehicle control (blue color) and after 1 week treatment with 25 mg amoxicillin (Amo)/kg body weight twice a day (red color) (top) and PC variance (middle and bottom). Amo was used as a positive control. A *p* value of less than 0.05 is considered a significant difference.

OG is used as a food additive, while it demonstrates antimicrobial activity. To assess whether oral consumption of OG has any impact on the gut microbiome, we performed microbiome assays by sequencing conserved 16S RNA gene fragments from individual DNA isolated from each mouse fecal sample before treatment and after oral administration of antibacterials twice a day for a week. Vehicle control (30% DMSO, 30% ethanol, and 40% TSB) was used as a negative control, and Amoxicillin (25 mg/kg body weight) was used as a positive control. The administration of vehicle control did not significantly affect the diversity of fecal microbiome in mice (*p* > 0.05; Figure 6A) and microbial community structure (see PC variance plots of middle and bottom Figure 6A). In contrast, the alpha-diversity of fecal microbiome was remarkably decreased 1 week after exposing the mice to 50 mg/kg OG twice a day for 7 days (*p* < 0.05; Figure 6B). Similarly, the microbial community structure was also altered by OG (see PC variance plots of middle and bottom Figure 6B). As expected, the positive control amoxicillin (25 mg/kg body weight) significantly changed the alpha-diversity and microbial community structure of fecal microbiome compared to vehicle control (*p* < 0.01; Figure 6C).

### 2.6. Oral Administration of OG Alters the Bacterial Species of Gut Microbiome in Mice

**Figure 7:**
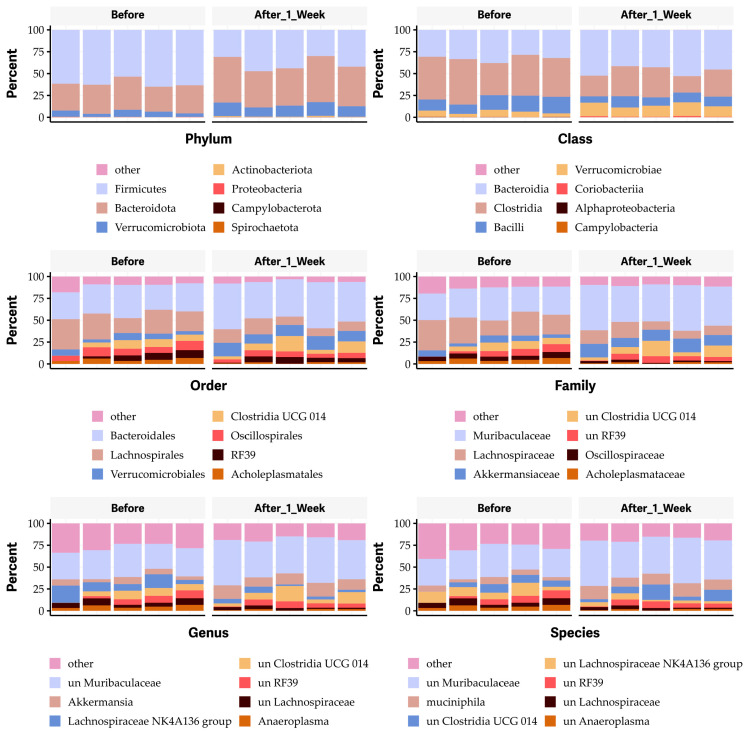
Impact of octyl gallate (OG) on the gut microbiome at the levels of phyla, class, order, family, genus, and species in mice.

To further elucidate which bacterial species of the gut microbiome are affected by the oral administration of OG, we performed a bioinformatic analysis. The results revealed that the vehicle control had no significant impact on the microbial community at any taxonomic level, including phylum, class, order, family, genus, and species (Figure S2). Notably, the oral administration of 50 mg/kg body weight OG obviously decreased the *Firmicutes* phylum, the *Clostridia* class (particularly the *Clostridia* vadinBB60 group), and the *Bacilli* class (Figure 7). It also decreased the unknown *Clostridia* vadinBB60 group while increasing the *Bacteroidetes phylum*, *Bacteroidia* class and order, *Verrucomicrobiae* class and order, and *Muribaculaceae* and *Akkermansiaceae* families, as well as *Mucispirillum schaedleri* and other unknown species (Figure 7). 

**Figure 8:**
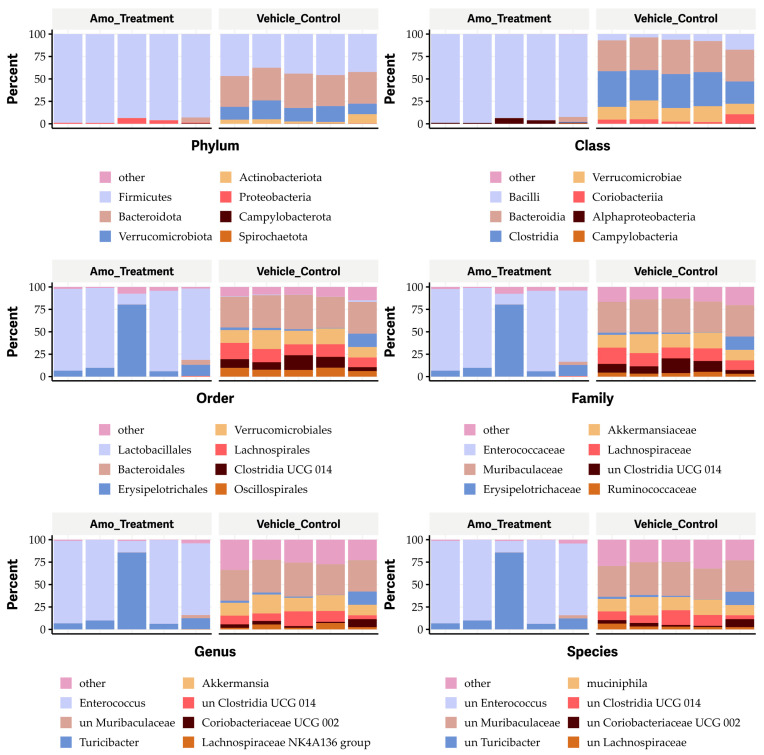
Impact of amoxicillin (Amo) on the phyla, classes, orders, families, genera, and species in the gut microbiome in mice.

As expected, oral administration of an antibiotic (25 mg amoxicillin/kg body weight twice a day for one week) resulted in dramatic changes in the gut microbiome, increasing the *Firmicutes* and *Proteobacteria* phyla (Figure 8), the *Bacilli* and alpha-*Proteobacteria* classes (Figure 8), the *Lactobacillales* and *Erysipelotrichales* orders (Figure 8), the *Enterococcaceae* and *Erysipelotrichaceae* families (Figure 8), and the *Enterococcus* and *Turicibacter* genera (Figure 8), as well as unknown species of *Enterococcus* and *Turicibacter* (Figure 8). Conversely, amoxicillin eliminated the *Actinobacteriota*, *Verrucomicrobiota*, and *Bacteroidota* phyla; the *Coriobacteria*, *Verrucomicrobiae*, *Clostridia*, and *Bacteroidia* classes; the *Bacteroidales, Verrucomicrobiales*, *Lachnospirales*, *Clostridia* UGG-014, *Oscillospirales*, and *Coriobacteriales* orders; the *Oscillospiraceae*, *Atopobiaceae*, and *Ruminococcaceae* families; and the unknown species of *Clostridia* UGG-014, *Lachnospiraceae*, *Akkermansiaceae*, *Muribaculaceae*, *Akkermansia*, and *Ruminococcaceae* (Figure 8). It also affected unknown species within the *Clostridia* UCG-014, *Coriobacteriaceae* UGG-002, and *Lachnospiraceae* families (Figure 8).

## 3. Discussion

Our findings support and extend previous research indicating that OG exhibits potent antibacterial and antibiofilm activities against a variety of bacterial strains, including *S. aureus* [11,18]. Our study demonstrated that OG is particularly effective against Gram-positive bacteria, including multi-drug-resistant strains such as HA-MRSA, CA-MRSA, LA-MRSA, *S. epidermidis*, *S. pyogenes*, and *B. subtilis*. With MICs ranging from 4 to 8 µg/mL and MBCs from 8 to 16 µg/mL, OG exhibits strong potential as a bactericidal agent (Table 1), which is consistent with previous reports [8,17]. Its potency against Gram-positive bacteria, including resistant strains, suggests that OG could be a valuable alternative or adjunct to traditional antibiotics, particularly in the face of rising antibiotic resistance [2,3,4,5]. However, OG’s limited activity against Gram-negative bacteria, such as *E. coli*, *K. pneumoniae*, and *P. aeruginosa* (MIC and MBC > 128 µg/mL) (Table 1), underscores the necessity for continued research into its mechanisms and potential modifications to enhance its spectrum of antimicrobial activity.

Further evaluation using kinetic studies revealed that OG inhibits *E. coli* growth in a dose-dependent manner, abolishing bacterial growth at concentrations above 128 µg/mL. Time-killing assays with HA-MRSA WCUH29 confirmed OG’s potent bactericidal action, demonstrating significant reductions in bacterial count within 30 min at the MBC level (16 µg/mL) and complete elimination after 3 h at higher concentrations (Figure 3). These results highlight OG’s rapid bactericidal effect compared to triclosan, which required a longer exposure period to achieve similar reductions. These findings suggest OG’s potential for fast-acting therapeutic applications, particularly for acute infections caused by Gram-positive bacteria.

Our results also suggest that OG may increase bacterial cell permeability in both Gram-positive and Gram-negative bacteria. This is evidenced by the significant increase in fluorescence intensity with the fluorescence probe PI in MRSA exposed to OG (8 µg/mL), consistent with previous findings [12] and its MICs against *S. aureus*. Similarly, exposure to OG (32 µg/mL) significantly increased the fluorescence intensity with the PI probe in *E. coli* in a dose-dependent manner, although the MIC of OG against *E. coli* is more than 128 µg/mL. OG’s varying efficacy against Gram-positive versus Gram-negative bacteria is likely due to obvious differences in their cell wall and membrane structure and efflux systems. *S. aureus*, with its thick peptidoglycan layer and absence of an outer membrane, relies on simpler systems like the MFS pump NorA and ABC transporter LmrA [27,28]. In contrast, *E. coli* has a complex cell envelope with both an inner and an outer membrane, requiring sophisticated systems like the RND family pump AcrAB-TolC, which spans both membranes to expel antibiotics [29]. Moreover, *E. coli* uses other systems, such as the MFS EmrB and ABC MacAB-TolC, to modulate its dual membrane structure [30,31]. 

OG is FDA-approved as a food additive in various food categories [32,33], with maximum permitted levels (MPLs) ranging from 25 mg/kg to 400 mg/kg depending on the product. For example, dehydrated milk and fats and oils with low water content have an MPL of 200 mg/kg, while chewing gum has a higher MPL of 400 mg/kg. Processed potato products have a lower MPL of 25 mg/kg. Other categories, including nut butter, fine bakery wares, non-heat-treated processed meat, seasonings, condiments, sauces, potato-, cereal-, flour-, or starch-based snacks, and food supplements, have MPLs between 200 mg/kg and 400 mg/kg [32,33,34]. Our study also demonstrated the low cytotoxicity of OG at 50 µg/mL (177.09 μM) in human HFF and A549 cells as well as low hemolytic activity at 200 µg/mL in sheep red blood cells. These findings indicate that further studies are needed to determine safe and effective dosing regimens that minimize cytotoxicity while maximizing antibacterial efficacy. 

Oral administration of OG remarkably altered the microbial diversity and community structure of gut microbiome in mice, suggesting the compound’s potential effects on gut health [35,36]. The control group, which received a vehicle, did not show significant changes in the gut microbiome, suggesting that the observed effects were specifically due to OG, consistent with previous findings [37,38]. Taxonomy analysis revealed a notable reduction in the *Firmicutes* phylum, particularly within the *Clostridia* and *Bacilli* classes, and an increase in the *Bacteroidetes* phylum and *Verrucomicrobiae* class. The decline in *Firmicutes*, including the *Clostridia* vadinBB60 group, contrasts with the enrichment of beneficial taxa, such as the *Muribaculaceae* and *Akkermansiaceae* families, and species like *Mucispirillum schaedleri*. These shifts in microbial populations could have significant implications for gut health, given the roles of these bacterial groups in metabolism, immune function, and disease.

The *Firmicutes* phylum is associated with short-chain fatty acid (SCFA) production, crucial for gut health [39]. A decrease in *Firmicutes*, particularly within the *Clostridia* and *Bacilli* classes, may indicate a shift in the gut’s metabolic capabilities. Studies suggest that a higher ratio of *Firmicutes* to *Bacteroidetes* is often associated with obesity and metabolic disorders [40]. Conversely, an increase in *Bacteroidetes* is generally considered beneficial, as these bacteria play a vital role in breaking down complex carbohydrates and fiber, thereby promoting SCFA production that benefits gut health and energy metabolism [41]. Members of the *Verrucomicrobiae* class, notably *Akkermansia muciniphila*, are known for their role in maintaining the gut mucus layer and enhancing gut barrier function. Increased abundance of *Verrucomicrobiae* has been linked to improved metabolic health and reduced inflammation [42]. Similarly, the *Muribaculaceae* family, within the *Bacteroidetes* phylum, and *Akkermansiaceae* family, particularly *Akkermansia muciniphila*, are associated with anti-inflammatory effects and the maintenance of gut homeostasis. *Mucispirillum schaedleri* contributes to modulating gut immunity and promoting gut health [43,44].

The in vitro antibacterial activity and the impact on bacterial cell permeability provide a mechanistic understanding of how OG can affect bacteria. These properties suggest that OG can selectively inhibit or kill certain bacteria, particularly Gram-positive pathogens. When OG is ingested, these antibacterial properties are exerted in the gut, leading to alterations in the gut microbiota composition and diversity. The reduction in microbial diversity and changes in community structure observed in the mouse gut microbiome following OG administration are consistent with the compound’s antibacterial effects. Thus, our study ties together in vitro observations with in vivo outcomes, demonstrating that OG’s antibacterial properties can extend beyond direct pathogen control to potentially modulating the gut microbiome.

Given OG’s efficacy as an antioxidant and antimicrobial agent in food preservation, its impact on the gut microbiome holds significant implications for food safety. The observed reduction in microbial diversity and shifts in community structure indicate the necessity for further investigation into the impact of OG on the gut microbiome and gut health, especially at FDA-approved doses.

Comparatively, the positive control, amoxicillin, drastically altered the gut microbiome, leading to decreased microbial diversity and changes in microbial community structure. The loss of microbial diversity has been associated with various health issues, including inflammatory bowel disease, obesity, and other metabolic disorders [45]. Our findings are consistent with previous reports indicating that diet and antibiotics can significantly affect the composition of the gut microbiome [46,47]. Amoxicillin dramatically increased the abundance of *Firmicutes* and *Proteobacteria* while eliminating *Actinobacteriota*, *Verrucomicrobiota*, and *Bacteroidota*, which are crucial for a healthy gut ecosystem [48]. This remarkable alteration underscores the broad-spectrum impact of traditional antibiotics, which can lead to dysbiosis and associated health issues [42]. Beneficial bacteria, such as *Akkermansia muciniphila* and *Ruminococcus* species, play key roles in maintaining gut barrier function and producing SCFAs, respectively [40,49,50]. Their reduction due to antibiotic use can compromise gut health [51,52]. The reduction of *Akkermansia* and *Ruminococcus* by amoxicillin highlights the importance of selective antimicrobial strategies to avoid disrupting these crucial microbial communities.

In conclusion, our study demonstrates that OG has a robust antibacterial activity against Gram-positive bacterial pathogens and has low cytotoxicity, and it selectively alters the gut microbiome, which may be beneficial for host health. These findings indicate that OG is a potential lead compound for developing new antibacterial agents that are effective yet minimize harm to the beneficial gut microbiome. 

## 4. Materials and Methods

### 4.1. Bacterial Strains and Growth Conditions

The *S. aureus* strains used in this study include the MRSA isolates USA200 HA-MRSA NRS383, USA400 HA-MRSA MW2, HA-MRSA WCUH29, USA700 MRSA NRS386, USA1000 MRSA NR483, USA1100 MRSA NRS484, CA-MRSA NRS194, NRS248, COL strains, USA300 CA-MRSA JE2, 923, 1371 strains, USA400 CA-MRSA NRS123, MRSA CFSa36, and LA-MRSA ST398, as well as the methicillin-sensitive *S. aureus* (MSSA) isolate MSA553, Newman and bovine RF122 strains (See Table 1). The *S. aureus* cells were cultured in Trypticase soy broth (TSB) at 37 °C with shaking. *S. pyogenes* was kindly provided by Dr. Jeffrey Hall. *E. coli* (ATCC 25922), *K. pneumoniae* (ATCC 13883), and *P. aeruginosa* (ATCC 27853) were cultured in *Luria broth* (LB).

### 4.2. Antibiotics and Chemical Compounds

Antibiotics, including Amoxicillin and Triclosan, and octyl gallate were purchased from Sigma-Aldrich, St. Louis, MO, USA (Catalog number 48700; Linear Formula: 3,4,5-(HO)_3_C_6_H_2_CO_2_(CH_2_)_7_CH_3;_ Molecular weight: 282.33). Fluorescent probes 1-N-phenylnaphthylamine and propidium iodide were purchased from Thermo Scientific, Waltham, MA, USA.

### 4.3. Eukaryotic Cell Culture 

Human foreskin fibroblast (HFF) cells (kindly provided by Dr. Roberta O’Connor) and human lung cancer epithelial A549 cells (ATCC) were cultured in RPMI 1640 medium and DMEM supplemented with 10% fetal bovine serum (FBS; Thermo Fisher Scientific, Waltham, MA, USA), respectively, as described [22]. Cultures of HFF and A549 cells were maintained in a medium containing penicillin (5 µg/mL) and streptomycin (100 µg/mL) (Thermo Fisher Scientific, Waltham, MA, USA). Assays were performed in RPMI 1640 or DMEM cell culture medium with different concentrations of octyl gallate (OG). 

### 4.4. MIC and MBC Assays

*S. aureus* strains were grown in Trypticase soy broth (TSB) at 37 °C overnight and were diluted to ~10^5^ CFU/mL in MHB for MIC assays with a 96-well microtiter format, as described previously [22]. Serial dilutions of the compounds were prepared in MHB broth in a final assay volume of 100 μL. Fifty microliters of 10^5^ CFU/mL bacteria was added to the serially diluted antibiotics. The MIC was the concentration at which the antibiotic prevented turbidity in the well after incubation for 18 h at 37 °C as described [22]. The MBC assay was conducted by dropping 10 μL of overnight culture (from the wells with 4×, 2×, and 1 × MIC in the 96-well plates of MIC assay) onto TSA. The MBC was the concentration at which the antibiotic killed the bacterial cells in the well after incubation for 18 h at 37 °C. The MIC and MBC assays were repeated at least three times, respectively.

### 4.5. Kinetic Time-Killing Assays

Kinetic time-killing assays for antimicrobial agents were conducted based upon the CLSI guidelines and the method described previously [22]. The HA-MRSA WCUH29 strain was grown into the early exponential phase in MHB at 37 °C with shaking at 225 rpm and exposed to different concentrations of antibacterial agents. The bacterial solution (50 μL) was collected from the culture at multiple time points and diluted in fresh TSB and plated onto TSA and incubated overnight at 37 °C for viable CFU. The time-killing assay was repeated at least three times.

### 4.6. Bacterial Cell Permeability Assays

The impact of OG on the membrane permeability of *S. aureus* and *E. coli* was examined following established protocols [12,53]. An overnight culture of *S. aureus* and *E. coli* was reinoculated into fresh TSB and LB media, respectively, and incubated to mid-log phase (OD_600nm_ = 0.5) at 37 °C with shaking at 225 rpm. The bacterial cells were harvested by centrifugation, washed once, and resuspended in an equal volume of 5 mM HEPES buffer containing 20 mM glucose, pH 7.2. The bacterial cells were then incubated with propidium iodide (10 μg/mL) for 10 min and exposed to different concentrations of OG (8, 16, and 32 μg/mL for *S. aureus*; 32, 64, or 128 μg/mL for *E. coli*). Fluorescence intensity was measured in a black 96-well plate using a BioTek Synergy II spectrophotometer (Agilent, Santa Clara, CA, USA) with 535 nm excitation and 590 nm emission. The level of permeability was adjusted by subtracting the fluorescence intensity of a negative control measured without bacterial cells. 

### 4.7. Cytotoxicity Assay with Human Foreskin Fibroblast (HFF) and Lung A549 Epithelial Cells

We performed cytotoxicity assays using both human HFF and A549 human lung cancer epithelial cells in triplicate experiments as described [22]. The cells were incubated in tissue culture flasks at 37 °C with 5% CO_2_ until a confluent monolayer was formed. Briefly, all cells were grown in 96-well plates to 90% confluence. To assess cytotoxicity, the monolayer cells were exposed to various doses of the tested compounds and incubated at 37 °C with 5% CO_2_ for 24 h. At the end of the experiment, cell viability was measured using the CellTiter 96 Aqueous Non-Radioactive Cell Proliferation Assay kit (Promega, Madison, MI, USA) according to the manufacturer’s instructions. DMSO was used as a negative control. Each experiment was repeated at least three times, and the percentage of cell survival relative to the negative control was calculated and statistically analyzed using Student’s *t*-test.

### 4.8. Hemolytic Activity Assay with Sheep Red Blood Cells

The hemolytic activity assay was conducted as described [54]. Briefly, fresh sheep red blood cells (RBCs) were collected by centrifugation (1000× *g*, 10 min), washed, and diluted 1:20 in PBS (pH 7.4). Aliquots (100 μL) of the RBC suspension (2 × 10^7^ cells/mL) were added to a U-bottomed 96-well plate containing different concentrations of OG (0, 50, 100, 200, 225, 250, 275, or 300 μg/mL) and incubated at 37 °C for 1 h. After incubation, the 96-well plate was centrifuged, and 100 μL aliquots of the supernatant were transferred to a new U-bottomed 96-well plate. Hemoglobin release due to RBC lysis was measured at 450 nm using a microplate reader. The positive control consisted of RBCs lysed with 0.5% saponin (1:1 vol/vol), while the negative control was RBCs suspended in PBS. The percentage of hemolysis was calculated using the following formula: [(A450 test sample − A450 negative control/(A450 positive control − A450 negative control)] × 100. 

### 4.9. Animal Experiments and Collection of Fecal Samples from Mice 

**Figure 9:**
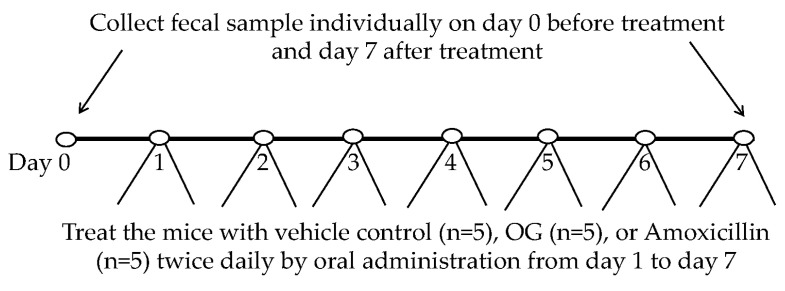
Diagram illustrating the schedule for collecting fecal samples and administering treatment for each group of mice.

CL57B/6 mice, aged 6 to 8 weeks, were used to investigate the impact of octyl gallate (OG) on the gut microbiome. Feces were collected from each mouse in both the vehicle control (n = 5) and OG group (n = 5). The cohort of mice (n = 5) was then treated via oral administration with either the vehicle control (n = 5) (30% DMSO, 30% ethanol, and 40% TSB), 50 mg/kg body weight OG (n = 5), or 25 mg/kg body weight amoxicillin (n = 5), twice daily for one week (Figure 9). Feces were collected individually after the final treatment dose and stored in a −80 °C freezer for subsequent total DNA purification from each fecal sample.

### 4.10. Total DNA Purification from Individual Fecal Sample and 16s RNA Sequencing and Microbiome Analysis

Total DNAs were extracted from the individual fecal sample using the DNeasy Blood and Tissue kits according to the manufacturer’s instructions (Qiagen, Valencia, CA, USA). The purified DNAs were then submitted to the University of Minnesota Genomic Center (UMGC) for 16S rRNA sequencing, which included quality control analysis, qPCR, and sequencing of the V4 region of the 16S rRNA. Negative controls included water blanks and negative extractions with each sequencing run.

16S rRNA Fastq sequences were trimmed for Illumina adapters and primers with Cutadapt version 4.6 [55]. Sequence files were further processed in DADA2 for quality using FilterandTrim (maxN = 0, maxEE = 2, truncQ = 2, rm.phix = TRUE); amplion sequence variant (ASV) table creation utilizing the DADA2 algorithm run with pseudo pooling. Chimeras were removed with the consensus method in removeBimeraDenovo. Taxonomy was assigned with DADA2′s assignTaxonomy and addSpecies using their maintained databases of rdp_train_set_18.fa.gz and rdp_species_assignment_18.fa.gz respectively.

Data Analysis: Principal coordinate analysis (PCoA) is a common method used to visualize and compare differences in microbial community structure [56]. The data analysis was done in R. Beta diversity using a customized CLR transform (https://github.com/trevorjgould/dada2_pipeline.git, accessed on 25 July 2024) followed by PCA. Alpha diversity simpson and shannon from vegan package, chao1 from OTUtable package. All plots used ggplot2, reshape2, and dlpyr for processing; cutadapt: http://journal.embnet.org/index.php/embnetjournal/article/view/200, accessed on 25 July 2024; GGplot2: H. Wickham. ggplot2: Elegant Graphics for Data Analysis. Springer-Verlag New York, 2016. Reshape2, https://github.com/hadley/reshape, accessed on 25 July 2024; Dplyr, https://dplyr.tidyverse.org, accessed on 25 July 2024; DADA2, https://benjjneb.github.io/dada2/tutorial.html, accessed on 25 July 2024; Bbmap, https://sourceforge.net/projects/bbmap/, accessed on 25 July 2024; and Vegan, https://cran.r-project.org/web/packages/vegan/vegan.pdf, accessed on 25 July 2024.

### 4.11. Data Analysis 

Independent samples were statistically analyzed using Student’s *t*-test with an alpha level ≤ 0.05 considered significant.

## Figures and Tables

**Table 1:** Antibacterial profiles of OG against both Gram-positive and Gram-negative bacteria.

Strain/Species	Discerption	OG MIC µg/mL	OG MBC µg/mL	Source or Ref.
*S. aureus*	MRSA			
WCUH29	HA-MRSA	8	16	NCIMB40771
NRS383	USA200 HA-MRSA	8	16	NARSA
MW2	USA400 HA-MRSA	8	16	[19]
NRS386	USA700 MRSA	8	16	Human isolate, NARSA
NRS483	USA1000 MRSA	4	8	Human isolate, NARSA
NRS484	USA1100 MRSA	16	32	Human isolate, NARSA
NRS194	CA-MRSA	8	16	NARSA
NRS248	CA-MRSA	8	16	NARSA
COL	CA-MRSA	8	16	[20]
JE2	USA300 CA-MRSA	8	16	BEI
923	USA300 CA-MRSA	8	16	[21]
1371	USA300 CA-MRSA	8	16	[22]
NRS123	USA400 CA-MRSA	8	16	NARSA
CFSa36	MRSA from CF patient	8	16	[23]
ST398	ST398 LA-MRSA	8	16	[24]
*S. aureus*	MSSA			
MSA553	ST30 MSSA	8	16	Dr. Patrick Schlievert
Newman	MSSA	8	16	[25]
RF122	ET3 ST151 bovine isolate	NA	16	[26]
Group A Streptococci				
*S. pyogenes*	Sore throat isolate	8	16	Dr. Jeffrey Hall
*Bacillus subtilis*		8	8	ATCC
Gram-negative bacteria				
*E. coli* ATCC 25922		>128	>128	ATCC
*A. baumannii* ATCC 19606		64	>64	ATCC
*K. pneumoniae* ATCC 13883		>128	>128	ATCC
*P. aeruginosa* ATCC 27853		>128	>128	ATCC

NARSA: Network of Antimicrobial Resistance in *Staphylococcus aureus*. BEI: Biodefense and Emerging Infections Research Resources Repository (BEI Resources).

## Data Availability

Data are contained within the article and Supplementary Materials.

## Supplementary Materials

The following supporting information can be downloaded at: https://www.mdpi.com/article/10.3390/antibiotics13080731/s1, Table S1: Raw Data; Figure S1: Determine the antibacterial activity (MIC) and MBC of OG against Gram-positive bacteria. Figure S2: Compare the impact of vehicle control (VC) on the gut microbiome before and after 1 week treatment at the levels of phyla, class, order, family, genus, and species.
