# Supplementary material for: Broad-Spectrum Antibacterial Activity of Antioxidant Octyl Gallate and Its Impact on Gut Microbiome
Source: Antibiotics (Basel). 2024 Aug 4;13(8):731. doi: 10.3390/antibiotics13080731 (PMC11350663; doi:10.3390/antibiotics13080731)
Supplement: Supplementary file 1 [file antibiotics-13-00731-s001.zip › antibiotics-3115675-supplementary figures-r2.pdf]

A

OG (μg/mL)

64
32
16
8
4
2
1
0

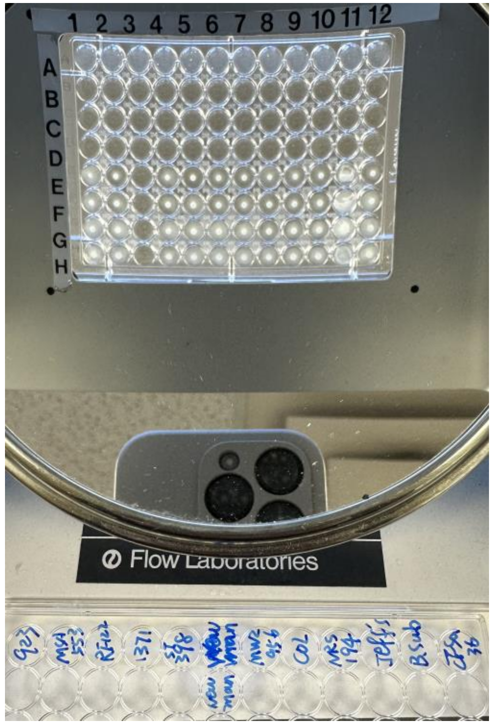

C

	MIC (μg/mL)	MBC (μg/mL)
CA-MRSA 923	8, 8, 8	16, 16, 16
MSA553	8, 8, 8	16, 16, 16
RF122	ND	16, 16, 16
USA300 CA-MRSA 1371	8, 8, 8	16, 16, 16
LA-MRSA ST398	8, 8, 8	8, 16, 16
Newman strain	8, 8, 8	16, 8, 16
HA-MRSA MW2 956	8, 8, 8	16, 16, 16
CA-MRSA COL	8, 8, 8	8, 16, 16
NRS194	8, 8, 8	8, 16, 16
S. pyogenes	8, 8, 8	16, 16, 16
B. Subtilis	8, 8, 8	16, 8, 8
CFSa36	8, 8, 8	8, 16, 16

B

MBC

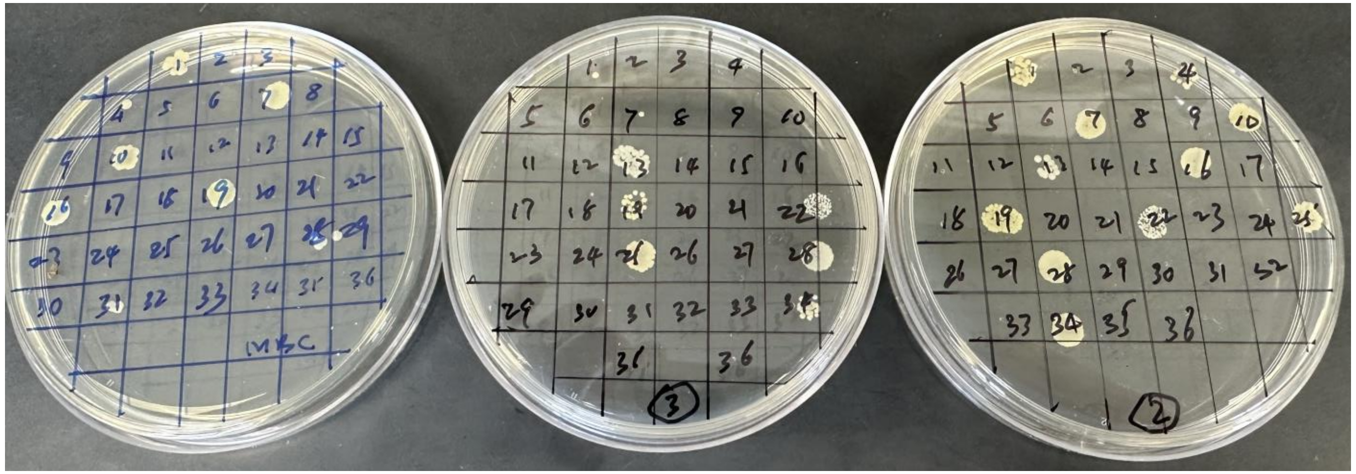

Figure S1. Determine the antibacterial activity (MIC) and MBC of OG against gram-positive bacteria. The values of MIC (A) and MBC (B) were determined in triplicates. (C) individual results for MIC and MBC. All assays were conducted as described in the Materials and Method section.

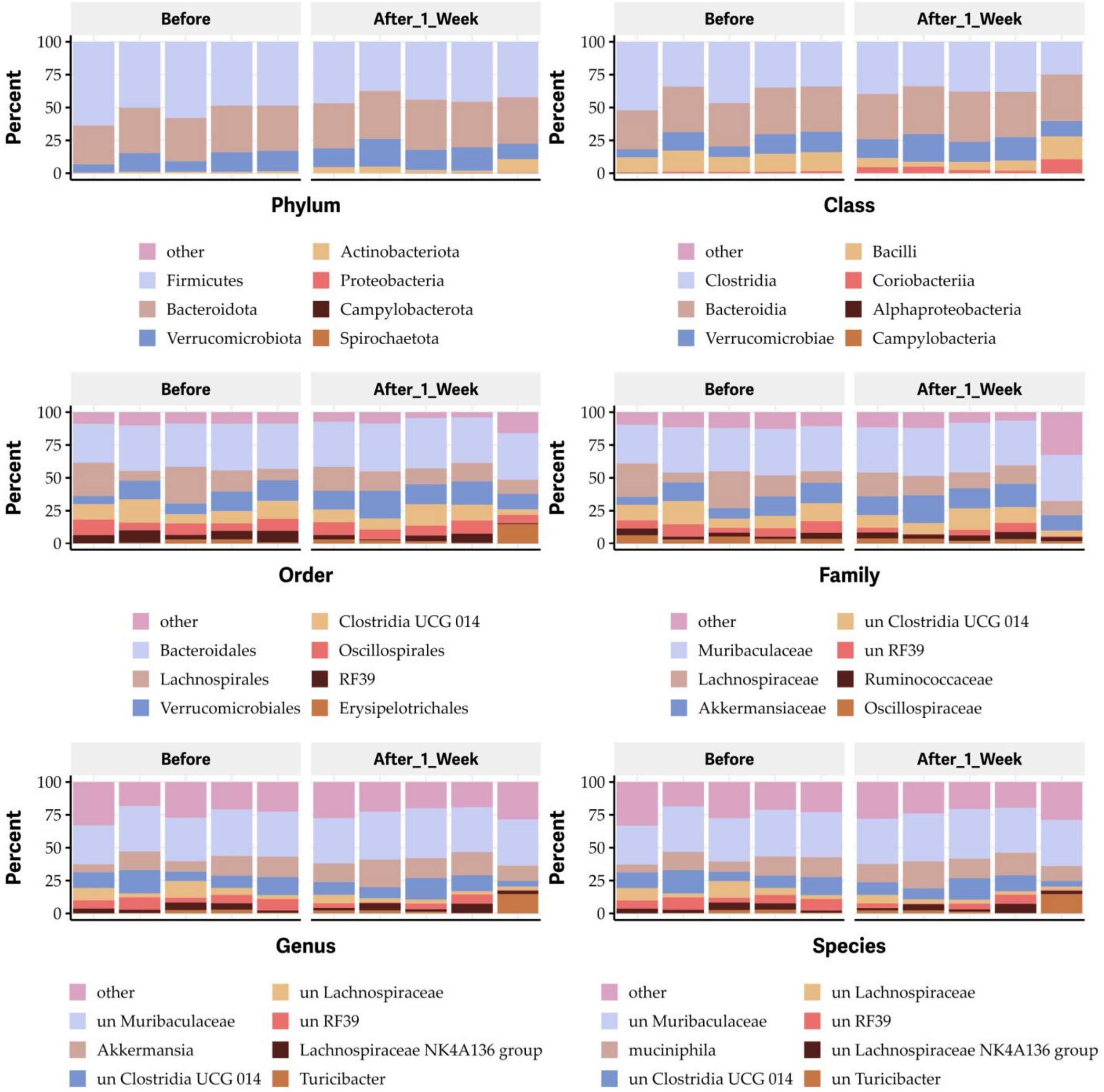

Figure S2. Compare the impact of vehicle control (VC) on the gut microbiome before and after 1 week treatment at the levels of phyla, class, order, family, genus, and species.
